# Comparison of OmpA Gene-Targeted Real-Time PCR with the Conventional Culture Method for Detection of *Acinetobacter baumanii* in Pneumonic BALB/c Mice

**DOI:** 10.29252/.23.2.159

**Published:** 2019-03

**Authors:** Niloofar Hassannejad, Abbas Bahador, Nasim Hayati Rudbari, Mohammad Hossein Modarressi, Kazem Parivar

**Affiliations:** 1 Dept. of Cell Biology, Science and Research branch, Islamic Azad University, Tehran, Iran;; 2 Dept. of Microbiology, Faculty of Medicine, Tehran University of Medical Sciences, Tehran, Iran;; 3 Dept. of Medical Genetics, Tehran University of Medical Sciences, Tehran, Iran

**Keywords:** Acinetobacter baumanii, OmpA, qRT-PCR Pneumonia, BALB/c mice

## Abstract

**Background:**

*Acinetobacter baumannii *is an important pathogen in health care and is responsible for severe nosocomial and community-acquired pneumonia. To design novel therapeutic agents, a mouse model for *A. baumannii *pneumonia is essential.

**Methods:**

We described a mouse model of *A. baumannii *using clinical and 19606R standard strains for developing a quantitative real-time PCR (qRT-PCR) for rapid identification of *A. baumannii* infection from lung tissues of BALB/c mice.

**Results:**

To infect the mice, three doses of bacteria (0.5 × 10^8^, 1 × 10^8^, and 1.5 × 10^8^ cfu/ml) were used. Lung tissues were cultured and compared with *ompA* gene. Clinical isolates had better positive results at day three with the highest dose than 19606 strain either in culture (4 versus 3) or in qRT-PCR (5 versus 4). However, qRT-PCR detection was 100%, the specificity was 70%, and the positive predictive value was 27%.

**Conclusion:**

The qRT-PCR detection of *A. baumannii *in the BALB/c mice model has a higher sensitivity than the culture method.

## INTRODUCTION

*Acinetobacter baumannii* is regarded as one of the most important opportunistic pathogens of increasing clinical importance over the course of the last three decades^[^^1^^,^^2^^]^. *A. baumannii *infection has recently emerged as a major cause of health care-associated (hospital- and community-acquired) infections worldwide. Pneumonia is the most frequent clinical presentation of *A. baumannii* infections^[^^3^^]^. This bacterial agent is identified as the fifth most common pathogen in Intensive care units (ICUs) in developed and developing countries^[^^2^^,^^4^^,^^5^^]^. The high rate of antibiotic resistance observed for *A. baumannii *isolates is attributed to its ability to employ a number of virulence factors such as OmpA^[^^6^^,^^7^^]^. OmpA is highly conserved among bacterial species, and in *A. baumannii*, it has been associated with a variety of interesting biological properties in *in vitro* model systems^[^^8^^,^^9^^]^. OmpA has also been associated with antimicrobial resistance in related pathogens^[^^10^^]^. The frequent existence of multidrug-resistant, extensive drug-resistant, and pandrug-resistant *A. baumannii* isolates revived the use of colistin, as an old polymyxin antibiotic^[^^11^^-^^14^^]^. Recently, colistin-resistant *A. baumannii* isolates are augmenting, which represents extremely alarming phenomenon^[^^15^^]^. Therefore, there is an urgent need for the development of novel therapeutic approaches.

Animal models are essential for the progress towards developing new therapeutics and vaccines and play fundamental roles in the evaluations of efficacy and safety of the new products before entering clinical trials. During the past years, the mice models of *A. baumannii *pneumonia have been developed and used widely^[^^3^^]^. In these models, several inbred strains of mice such as C57BL/6 and BALB/c as well as A/J strains have been used. Although these strains are likely to demonstrate a more homogeneous response to infection, they may not exactly mirror the variable responses that may take place in humans^[^^16^^-^^21^^]^. To recognize the infected mice and verify the production of infectious models, researchers apply diagnostic methods. Current diagnostic techniques rely on culture-based approaches, which are time-consuming and dependent on individual interpretation. In addition, the sensitivity of culture method is not high enough^[^^22^^]^. Nowadays, the real-time PCR is being used for sensitive detection of different viral^[^^23^^]^ and bacterial infections^[^^24^^]^, which improves the early detection of these diseases. 

The aim of this study was to optimize a quantitative real-time PCR (qRT-PCR) method for rapid identification of *A. baumannii* in infected lung tissues of BALB/c mice using a clinical isolate and the strain 19606 of *A. baumannii*.

## MATERIALS AND METHODS

**Bacterial strains and culture conditions**

A CR-XDR *A. baumannii* isolated from burn-wound infection was used in this study^[25]^. This strain is resistant to amikacin, cefepime, ceftazidime, ciprofloxacin, colistin, gentamicin, imipenem, levofloxacin, minocycline, tetracycline, tobramycin, and trimethoprim-sulfamethoxazole, but it is susceptible to ampicillin-sulbactam (Table 1). The phenotype of *A. baumannii* is defined as XDR, which is consistent with the International Expert Proposal for Interim Standards Guidelines^[^^26^^]^. *A. baumannii* ATCC 19606R was incorporated in the study as a colistin and an imipenem-susceptible reference strain (Table 1). Fresh brain heart infusion broth (Merck, Darmstadt, Germany) bacterial cultures, in an aerobic atmosphere in the logarithmic growth phase (4–5 hours) at 37 °C, were adjusted to a concentration of 1.0 × 10^6 ^colony forming units (CFU)/mL, as verified by both spectrophotometry (OD_600_ 0.01–0.02 nm) and colony counting^[^^27^^]^.

**Table 1:** Two multidrug-resistant *A. baumannii* strains were used and the minimum inhibitory concentration (MIC) of 13 antimicrobial drugs for these strains were determined

**Antibiotics**	**Types of strain/MIC (μg/ml)**	
	**Clinical isolate**	**19606R (standard) strain**
Β-Lactamase inhibitors Ampicillin-sulbactam	4/2 (S)	32/16 (R)

Cephems		
Ceftazidime	64 (R)	32 (R)
Cefepime	64 (R)	32 (R)
		
Carbapenems		
Imipenem	16 (R)	2 (S)

Lipopeptides		
Colisitin	16 (R)	2 (S)
		
Aminoglycosides		
	32 (R)	16 (R)
Amikacin	128 (R)	64 (R)
Tobramycin	32 (R)	16 (R)
		
Tetracyclines		
Minocycline	32 (R)	16 (R)
Tetracycline	32 (R)	16 (R)
		
Fluoroquinolones		
Ciprofloxacin	8 (R)	4 (R)
Levofloxacin	16 (R)	8 (R)
		
Folat pathway inhibitors		
Trimethoprim-sulfamethoxazole	8/152 (R)	4/76 (R)

(R) resistant; (S) susceptible

***A. baumannii*** **isolate confirmation**

The isolate was identified as *A. baumannii *using API 20NE system (bioMérieux, Marcy-l'Etoile, France) and was later confirmed by the detection of *bla*OXA-51- PCR, to differentiate between *Acinetobacter calcoaceticus* and *Acinetobacter genomic species*^[^^28^^,^^29^^]^. The clinical isolate was stored at -20 °C in CRYOBANK™ (Copan Diagnostics Inc., Canada) until further use.

**Mice**

Eight- to 12-week-old, specific-pathogen free, male or female BALB/c mice, with 25–35 g in weight, were purchased from the *Pasteur Institute *of Iran (Karaj, *Iran*). Animals had free access to food and water except during experimental procedures. All studies were performed in accordance with the institutional ethical guidelines for the care and use of laboratory animals, and the protocol was approved by Tehran University of Medical Sciences, Tehran, Iran^[^^30^^]^. 

**Intranasal** ***A. baumannii*** **inoculation**

For intranasal inoculation, fresh inocula were prepared for each experiment from frozen stocks of the *A. baumannii *isolate as previously mentioned by van Faassen *et al*.^[^^31^^]^. Mice were anesthetized by intraperitoneal (i.p.) injection of 12.5 mg/kg (5 µl) xylazine and 80 mg/kg (25 µl) ketamine and then inoculated intranasally with appropriate numbers of various *A. baumannii *isolates in 50 μl of saline. For evaluation of infection using real-time PCR, 180 mice were assigned to four groups (5 mice in each group) using three doses (0.5 × 10^8^, 1 × 10^8^, and 1.5 × 10^8^ cfu/ml) of bacteria in 50 µl for three days.

**Conventional bacterial culture of pneumonic mice lung**

Five mice of each group were sacrificed 24 hours after days 1, 2, and 3 post inoculation. The lung (about 0.36 g) was removed and segmented and then homogenized under the sterile condition. Next, 1 ml saline was added to the homogenized tissue, and 100 μl was cultured on Muller-Hinton agar (MHA) and then incubated at 37 °C for one day. One part of tissue was removed for qRT-PCR, and the rest was used for the conventional culture. 

**Evaluation of the** ***A. baumannii*** **OmpA gene expression by qRT-PCR**

**DNA extraction from lung tissue**

A commercial kit (DNeasy Blood and Tissue Kit, Qiagen Inc., Valencia, CA, USA) was used in accordance with manufacturer’s recommendations to extract DNA. About 4 g of each tissue was dissected using a sterile scalpel blade and placed in a stomacher bag containing 20 ml of Earle's balanced salt solution. The sample was homogenized with a blender at high speed for 60 s. The resultant supernatant was stored at -20 °C until it was used for DNA extraction. To extract DNA, 100 μl of liquid homogenate was mixed with 180 μl of ATL buffer (Tissue Lysis Buffer), supplied with the kit, and then the kit’s spin-column protocol for “purification of total DNA from animal tissues” was used.

**Primers**

Primers used for qRT-PCR, previously published by McConnell *et al.*^[^^32^^]^ are as follows: OmpA-F (TCTTGGTGGTCACTTGAAGC) and OmpA-R (ACTCTTGTGGTTGTGGAGCA) as well as 16sr RNA-F (ACTCCTACGGGAGGCAGCAGT) and 16sr RNA-R (TATTACCGCGGCTGCTGGC). Sequences were checked for specificity using NCBI primer BLAST (http://www.ncbi.nlm.nih.gov/tools/primer-blast/). All primers were optimized using temperature gradients, and the PCR products were visualized on 1% agarose gel electrophoresis. The equivalent Tm value for *OmpA* gene (Tm: 79.4 ± 0.4 °C) was also detected when the positive control strain (*A. baumanii*; ATCC 19606 standard strain) was tested with qRT-PCR. 

**qRT-PCR **

All real-time PCRs were performed on an ABI 7300 qRT-PCR detection system (Applied Biosystems, Foster City, CA, USA). Real-time qPCR amplification was carried out using the *Qiagen Rotor-Gene SYBR Green Kit*. A standard curve (using at least 5 standards) as well as positive, negative, and no template controls were included in every run. For SYBR green-based reactions, the conditions were as follows: 10 min at 95 °C, followed by 40 cycles of 5 s at 95 °C and 10 s at 60 °C. At the end of each reaction, a melting curve (from 50 to 99 °C) was performed. In order to rule out the false-negative results due to DNA losses during DNA extraction or the presence of PCR inhibitor in the template, human herpes virus 6 (HHV6) DNA (obtained from the Department of Virology, Tehran University of Medical Sciences, Tehran, Iran) was sed as an internal control. Mice were scarified at days 1, 2, and 3 post inoculation with three sub-lethal concentrations of the bacteria (0.5 × 10^8^, 1 ×10^8^, and 1.5 × 10^8 ^CFU/ml), and the tissue was evaluated with qRT-PCR OmpA expression.

**Fig. 1:**
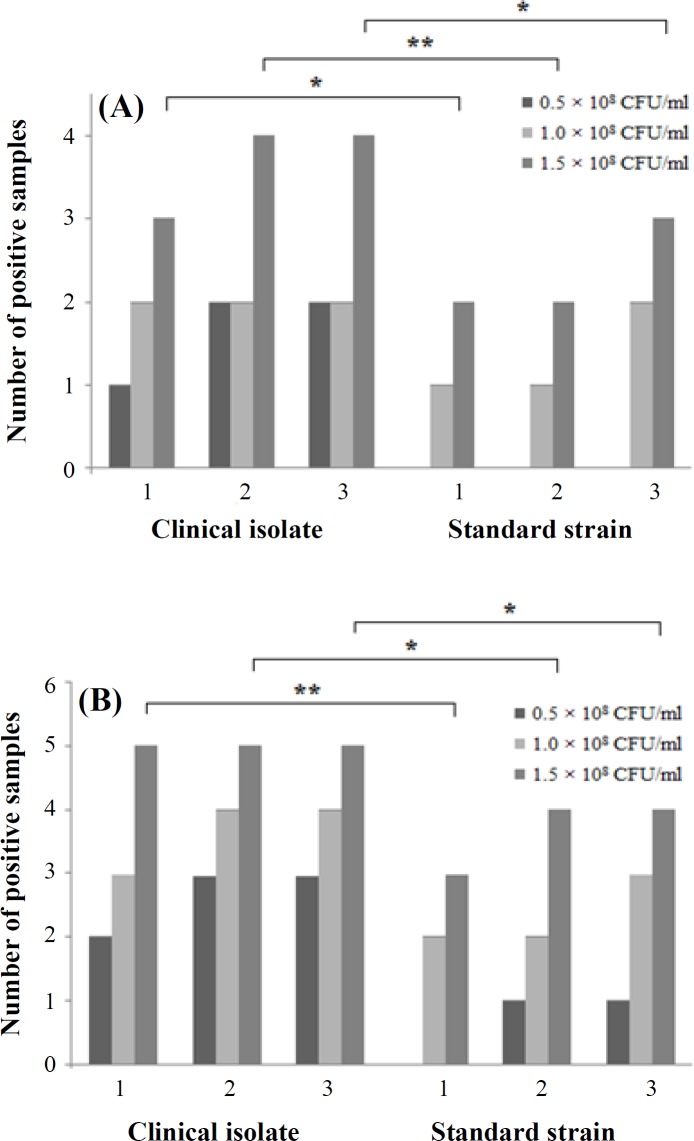
Positive results of *A.baumannii *in the samples of lung tissues of pneumonic mice. Mice were inoculated with three different doses of clinical isolate and standard strain (19606R), and numbers of colonies and *OmpA* genes were measured on days 1, 2, and 3 post inoculation. (A); Number of colonies detected in the traditional tissue culture. (B); Number of *OmpA* gene detected by qRT-PCR directly from lung tissue. Twenty mice were used for each dose of bacteria, and five mice were used in each group (total 180 mice). ^*^ and ^**^ represent *p* < 0.05 and *p* < 0.01, respectively

**Statistical analysis**

Data were presented as means ± standard deviation (SD) for each group. Differences were assessed by student’s *t*-test by one-way ANOVA. Differences were considered statistically significant when *p* ≤ 0.05.

## RESULTS

**Establishment of qRT-PCR assays**

The linearity and limits of detection of the assays were determined with serial 10-fold dilutions of ATCC 19606R DNA from 10^1^ to 10^6^ copies/μL. The limit of detection for the target DNA was 10 copies per 20 μL reaction volume. The assays correlated well for OmpA (r^2^ = 0.994). Intra- and inter-assay repeatability was tested in triplicate for each dilution within the same run, and each concentration was repeated three different times to assess the reproducibility of the qRT-PCR assays. The coefficient of variation of intra-assay and inter-assay were low and in the ranges of 0.06%–0.48% and 0.08%–4.40%, respectively. Intra- and inter-assay coefficient of variation of less than 4.2% confirmed the high repeatability of the assays. Samples were considered positive if a threshold cycle was reached during the 35 cycles. 

**Comparison of conventional bacterial culture results with qRT-PCR**

Since *OmpA* sequence of different strains of *A. baumannii* are similar, the standard curve was used to determine the existence of bacterial genomic DNA in the lung tissue. On third day post infection, the lungs of the infected mice in conventional culture showed the highest positive number when the highest inoculation dose (1.5 × 10^8^ CFU/ml) was used; however, clinical isolate had more positive results than the standard strain (Fig. 1A). The direct qRT-PCR had most sensitivity and rapid detection of *OmpA* gene expression. The number of positive results in direct qRT-PCR was higher in comparison with conventional cultures, in all three bacterial doses on each day after inoculation (Fig. 1B).

## DISCUSSION

This report is the first observational study to assess the clinical utility of qRT-PCR on specimens from the respiratory tract of a mouse model for detection of *A. baumannii*. The previous results from patient samples in the ICUs demonstrated that *A. baumannii *was identified on 8.7% of cases using bacterial culture^[^^33^^]^. In our study, we showed that the positive bacterial culture in three different doses were 0-80% of bacteria in the lung of mice inoculated with *A. baumannii*, whereas qRT-PCR could detect a higher rate (10-100%) of the bacteria (as OmpA). We also demonstrated a high sensitivity and negative predictive value of direct qRT-PCR detection for identification of

*A. baumannii.* The results revealed that in both methods (conventional culture and qRT-PCR), the highest dose (1.5 × 10^8^ CFU/ml) of the bacterial inoculation had the best positive outcomes in all three days post inoculation, especially in relation to the clinical isolate of *A. baumannii*^[^^22^^]^.

Using positive culture of *A. baumannii*, as the gold standard, the sensitivity of qRT-PCR detection was 100%, the specificity was 70%, the positive predictive value was 27%, and the negative predictive value was 100% among the samples collected from *A. baumannii* pneumonic mice. We observed a high frequency of samples that were negative by the conventional bacterial culture but positive by qRT-PCR. One possibility is that some Acinetobacter strains that are detected by qRT-PCR could not able to grow on Acinetobacter medium^[^^24^^]^. The application of qRT-PCR was limited by the low positive predictive value for the following possibilities. First, negative culture results could have been obscured by immune system of mice in some cases. As such, the dead bacterial remnants would fail to grow on culture, but the qRT-PCR detects the presence of genomic DNA in the samples. Second, more sensitive nature of qRT-PCR detection might result in recognizing a small number of *A. baumannii*, which might not be detected in culture^[^^23^^]^. Third, the PCR might be attributed to a false-positive results, possibly indicating the existence of bacterial DNA that is not associated with viable bacteria^[^^32^^]^. However, the inclusion of a negative PCR control minimized this possibility. Fourth, more sensitive qRT-PCR might detect a small number of *A. baumannii*, which are not responsible agent for pneumonia.

In conclusion, our study indicated that qRT-PCR is a sensitive method for diagnosis of *A. baumannii* in pneumonic animal models. However, further studies are required to probe the potential role of this approach. 

## References

[1] Hood MI, Jacobs AC, Sayood K, Dunman PM, Skaar EP (2010). Acinetobacter baumannii increases tolerance to antibiotics in response to monovalent cations. Antimicrobial agents and chemotherapy.

[2] Peleg AY, Seifert H, Paterson DL (2008). Acinetobacter baumannii: emergence of a successful pathogen. Clinical microbiology reviews.

[3] McConnell MJ, Actis L, Pachón J (2013). Acinetobacter baumannii: human infections, factors contributing to pathogenesis and animal models. FEMS microbiology reviews.

[4] King LB, Pangburn MK, McDaniel LS (2013). Serine protease PKF of Acinetobacter baumannii results in serum resistance and suppression of biofilm formation. The Journal of infectious diseases.

[5] Fouad M, Attia AS, Tawakkol WM, Hashem AM (2013). Emergence of carbapenem-resistant Acinetobacter baumannii harboring the OXA-23 carbapenemase in intensive care units of Egyptian hospitals. International journal of infectious diseases.

[6] Smani Y, Fàbrega A, Roca I, Sánchez-Encinales V, Vila J, Pachón J (2014). Role of OmpA in the multidrug resistance phenotype of Acinetobacter baumannii. Antimicrobial agents and chemotherapy.

[7] Fournier PE, Vallenet D, Barbe V, Audic S, Ogata H, Poirel L, Richet H, Robert C, Mangenot S, Abergel C, Nordmann P, Weissenbach J, Raoult D, Claverie JM (2006). Comparative genomics of multidrug resistance in Acinetobacter baumannii. PLoS genetics.

[8] Magnet S, Courvalin P, Lambert T (2001). Resistance-nodulation-cell division-type efflux pump involved in aminoglycoside resistance in Acinetobacter baumannii strain BM4454. Antimicrobial agents and chemotherapy.

[9] Sugawara E, Nikaido H (2012). OmpA is the principal nonspecific slow porin of Acinetobacter baumannii. Journal of bacteriology.

[10] Kim SW, Choi CH, Moon DC, Jin JS, Lee JH, Shin JH, Kim JM, Lee YC, Seol SY, Cho DT, Lee JC (2009). Serum resistance of Acinetobacter baumannii through the binding of factor H to outer membrane proteins. FEMS microbiology letters.

[11] Beceiro A, Llobet E, Aranda J, Bengoechea JA, Doumith M, Hornsey M, Dhanji H, Chart H, Bou G, Livermore DM, Woodford N (2011). Phosphoethanolamine modification of lipid A in colistin-resistant variants of Acinetobacter baumannii mediated by the pmrAB two-component regulatory system. Antimicrobial agents and chemotherapy.

[12] Nasnas R, Saliba G, Hallak P (2009). The revival of colistin: an old antibiotic for the 21st century. Pathologie Biologie (Paris).

[13] Lee HJ, Bergen PJ, Bulitta JB, Tsuji B, Forrest A, Nation RL, Jian L (2013). Synergistic activity of colistin and rifampin combination against multidrug-resistant Acinetobacter baumannii in an in vitro pharmacokinetic/ pharmacodynamic model. Antimicrobial agents and chemotherapy.

[14] Cai Y, Chai D, Wang R, Liang B, Bai N (2012). Colistin resistance of Acinetobacter baumannii: clinical reports, mechanisms and antimicrobial strategies. Journal of antimicrobial chemotherapy.

[15] Valencia R, Arroyo LA, Conde M, Aldana JM, Torres MJ, Fernández-Cuenca F, Garnacho-Montero J, Cisneros JM, Ortíz C, Pachón J, Aznar J (2009). Nosocomial outbreak of infection with pan-drug-resistant Acinetobacter baumannii in a tertiary care university hospital. Infection control and hospital epidemiology.

[16] Crandon JL, Kim A, Nicolau DP (2009). Comparison of tigecycline penetration into the epithelial lining fluid of infected and uninfected murine lungs. Journal of antimicrobial chemotherapy.

[17] Joly-Guillou ML, Wolff M, Pocidalo JJ, Walker F, Carbon C (1997). Use of a new mouse model of Acinetobacter baumannii pneumonia to evaluate the postantibiotic effect of imipenem. Antimicrobial agents and chemotherapy.

[18] Elhosseiny NM, Amin MA, Yassin AS, Attia AS (2015). Acinetobacter baumannii universal stress protein A plays a pivotal role in stress response and is essential for pneumonia and sepsis pathogenesis. International journal of medical microbiology.

[19] Braunstein A, Papo N, Shai Y (2004). In vitro activity and potency of an intravenously injected antimicrobial peptide and its DL amino acid analog in mice infected with bacteria. Antimicrobial agents and chemotherapy.

[20] Koomanachai P, Kim A, Nicolau DP (2009). Pharmacodynamic evaluation of tigecycline against Acinetobacter baumannii in a murine pneumonia model. Journal of antimicrobial chemotherapy.

[21] Song JY, Cheong HJ, Lee J, Sung AK, Kim WJ (2009). Efficacy of monotherapy and combined antibiotic therapy for carbapenem-resistant Acinetobacter baumannii pneumonia in an immunosuppressed mouse model. International journal of antimicrobial agents.

[22] Deschaght P, De Baere T, Van Simaey L, VAn Daele S, De Baets F, De Vos D, Pirnay JP, Vaneechoutte M (2009). Comparison of the sensitivity of culture, PCR and quantitative real-time PCR for the detection of Pseudomonas aeruginosa in sputum of cystic fibrosis patients. BMC microbiology.

[23] Fothergill JL, Ledson MJ, Walshaw MJ, McNamara PS, Southern KW, Winstanley C (2013). Comparison of real time diagnostic chemistries to detect Pseudomonas aeruginosa in respiratory samples from cystic fibrosis patients. Journal of cystic fibrosis.

[24] Gadsby N, McHugh M, Russell C, Mark H, Conway Morris A, Laurenson IF, Hill AT, Templeton KE (2015). Development of two real-time multiplex PCR assays for the detection and quantification of eight key bacterial pathogens in lower respiratory tract infections. Clinical microbiology and infection.

[25] Bahador A, Raooﬁan R, Farshadzadeh Z, Beitollahi L, Khaledi A, Rahimi S, Mokhtaran M, Mehrabi Tavana A, Esmaeili D (2015). The prevalence of ISAba1 and ISAba4 in Acinetobacter baumannii species of different international clone lineages among patients with burning in Tehran, Iran. Jundishapur journal of microbiology.

[26] Magiorakos AP, Srinivasan A, Carey RB, Carmeli Y, Falagas ME, Giske CG, Harbarth S, Hindler JF, Kahlmeter G, Olsson-Liljequist B, Paterson DL, Rice LB, Stelling J, Struelens MJ, Vatopoulos A, Weber JT, Monnet DL (2012). Multidrug‐resistant, extensively drug‐resistant and pandrug‐resistant bacteria: an international expert proposal for interim standard definitions for acquired resistance. Clinical microbiology and infection.

[27] Dai T, Murray CK, Vrahas MS, Baer DG, Tegos GP, Hamblin MR (2012). Ultraviolet C light for Acinetobacter baumannii wound infections in mice: Potential use for battlefield wound decontamination?. The journal of trauma and acute care surgery.

[28] Higgins PG, Lehmann M, Wisplinghoff H, Seifert H (2010). gyrB multiplex PCR to differentiate between Acinetobacter calcoaceticus and Acinetobacter genomic species 3. Journal of clinical microbiology.

[29] Wang X, Chen T, Yu R, Lü X, Zong Z (2013). Acinetobacter pittii and Acinetobacter nosocomialis among clinical isolates of the Acinetobacter calcoaceticus-baumannii complex in Sichuan, China. Diagnostic microbiology and infectious disease.

[30] Harris G, Kuo Lee R, Lam CK, Kanzaki G, Patel GB, Xu HH, Chen W (2013). A mouse model of Acinetobacter baumannii-associated pneumonia using a clinically isolated hypervirulent strain. Antimicrobial agents and chemotherapy.

[31] Van Faassen H, KuoLee R, Harris G, Zhao X, Conlan JW, Chen W (2007). Neutrophils play an important role in host resistance to respiratory infection with Acinetobacter baumannii in mice. Infection and immunity.

[32] McConnell MJ, Pérez-Ordóñez A, Pérez-Romero P, Valencia R, Lepe JA, Vázquez-Barba I, Pachón J (2012). Quantitative real-time PCR for detection of Acinetobacter baumannii colonization in the hospital environment. Journal of clinical microbiology.

[33] Chiang MC, Kuo SC, Chen YC, Lee YT, Chen TL, Fung CP (2011). Polymerase chain reaction assay for the detection of Acinetobacter baumannii in endotracheal aspirates from patients in the intensive care unit. Journal of microbiology, immunology and infection.

